# Embedding the Bioactive Agent in Dye Structure for Development of Environmentally Sustainable Bioactive Textiles

**DOI:** 10.3390/biomimetics11070477

**Published:** 2026-07-08

**Authors:** Anum Nosheen, Munir Ashraf, Azam Ali, Muhammad Zaman Khan, Aiyeshah Alhodaib

**Affiliations:** 1 Functional Textiles Research Group, School of Engineering & Technology, National Textile University, Faisalabad 37610, Pakistan; nosheenabbas735@gmail.com; 2 School of Culture and Design, Clothing Technology, HTW Berlin–University of Applied Sciences for Technology and Economics, 12459 Berlin, Germany; munir.ashraf@htw-berlin.de; 3 Department of Material Engineering, Faculty of Textile Engineering, Technical University of Liberec, 461 17 Liberec, Czech Republic; azam.ali@tul.cz; 4 Department of Physics, College of Science, Qassim University, Buraydah 51452, Qassim, Saudi Arabia; ahdieb@qu.edu.sa

**Keywords:** novel reactive dye, sustainable bioactive textiles, non-leaching, durability, colorfastness properties, UV protection

## Abstract

The growing demand for durable and environmentally sustainable bioactive textiles has created a need for functionalization strategies that minimize the release of active agents during use and laundering. In this study, a novel chloroxylenol-functionalized vinyl sulfone reactive dye was synthesized through the coupling of chloroxylenol with diazotized para-ester and characterized using FTIR, UV–Vis, ^1^H-NMR, and ^13^C-NMR spectroscopy. The synthesized dye was applied to cotton fabric through an exhaust dyeing process, enabling simultaneous coloration and biofunctionalization in a single step. The dye exhibited high substantivity toward cotton, achieving dye exhaustion and fixation values of 95% and 91%, respectively. The dyed fabric demonstrated excellent antibacterial activity against *Staphylococcus aureus* (99.99%) and *Escherichia coli* (94%), antiviral activity of 87%, and antifungal activity of 86% before laundering. After 20 laundering cycles, antibacterial activity remained at 96% against *S. aureus* and 91% against *E. coli*, while antiviral and antifungal activities remained at 83% and 82%, respectively, confirming the durability of the bioactive functionality. Optical density measurements further verified substantial bacterial growth inhibition, whereas MTT assays using L929 fibroblasts demonstrated acceptable biocompatibility with cell viability exceeding 80% at the highest tested concentration. The dyed fabrics also exhibited excellent ultraviolet protection (UPF 119) with UVA and UVB blocking efficiencies of 99.35% and 98.98%, respectively, together with good colorfastness properties. Furthermore, UV–Vis analysis of the washing liquor indicated negligible dye release under the investigated laundering conditions. These findings demonstrate an effective and sustainable one-step strategy for producing durable multifunctional bioactive textiles while reducing processing steps and minimizing the potential release of active agents during use.

## 1. Introduction

The growing demand for hygienic and infection-resistant materials, particularly after the COVID-19 pandemic, has accelerated the development of bioactive textiles for healthcare, personal protection, and hygiene applications [1]. Such textiles are designed to inhibit the growth and transmission of pathogenic microorganisms, thereby reducing the risk of cross-contamination and hospital-acquired infections. However, achieving durable antimicrobial performance while maintaining environmental sustainability remains a major challenge. Conventionally, bioactive textiles are produced by applying antimicrobial agents onto the fabric surface through coating or finishing treatments. A wide variety of inorganic and organic agents, including metallic nanoparticles, halogenated phenols, and quaternary ammonium compounds, have been employed for this purpose [2]. Among these, silver-based systems are the most widely used owing to their broad-spectrum antimicrobial activity. Nevertheless, the release of silver species during laundering has raised concerns regarding toxicity, bioaccumulation, and adverse effects on both humans and aquatic ecosystems [3]. Similarly, halogenated phenols such as triclosan have been associated with genotoxicity, endocrine disruption, and cytotoxic effects on aquatic organisms [4], whereas exposure to quaternary ammonium compounds has been linked to respiratory disorders, including asthma and chronic obstructive pulmonary disease [5]. Natural dyes and plant-derived extracts have also been investigated as sustainable alternatives for developing bioactive textiles [6]. Although these systems offer reduced toxicity, their practical implementation is limited by low durability, poor reproducibility, lengthy extraction procedures, and restricted availability of raw materials. More recently, metal nanoparticles such as Ag-NPs, Zn-NPs, Si-NPs, and Ti-NPs have been incorporated into textile substrates to impart antibacterial, antifungal, and antiviral functionalities [7]. Despite their effectiveness, these surface-treated systems often exhibit poor laundering durability, resulting in continuous leaching of active agents into wastewater during washing cycles [8]. Such a release not only contributes to environmental contamination but may also disturb microbial populations involved in biological wastewater treatment processes [9]. Furthermore, exposure to sub-inhibitory concentrations of antimicrobial agents can promote the emergence of resistant microorganisms [10]. Consequently, most commercially available bioactive textiles suffer from a limited service life and loss of functionality after repeated laundering [2]. To overcome these limitations, researchers have explored integrated dyeing and finishing approaches in which the bioactive functionality is chemically incorporated into reactive dye molecules. Zhang et al. reported a one-step dyeing and antimicrobial finishing process for cotton fabrics using reactive dyes [11]. Similarly, Mohammad et al. synthesized triazine-based reactive dyes containing glutaraldehyde and terephthalaldehyde as bioactive moieties [12], while Sagheer et al. developed antimicrobial reactive dyes using sulfonamide derivatives [13]. These approaches improve washing durability and reduce water and chemical consumption due to the formation of strong covalent bonds between the reactive dye and cellulosic fibers. Nevertheless, several reported systems require prolonged contact times to achieve satisfactory antimicrobial performance, thereby limiting their practical applicability. Despite recent advances in sustainable coloration and finishing technologies, preventing the release of bioactive agents from textiles during laundering remains a significant challenge. Therefore, the present study aimed to develop a durable and potentially more environmentally favorable approach than conventional multi-step finishing approaches. A novel vinyl sulfone-based reactive dye was synthesized using chloroxylenol as the coupling component with diazotized para-ester. To the best of our knowledge, chloroxylenol has not previously been utilized as a precursor for the synthesis of reactive dyes intended for multifunctional bioactive textiles [7]. The central hypothesis of this study is that covalently incorporating chloroxylenol into the molecular structure of a vinyl sulfone reactive dye, rather than applying it as a conventional post-finishing agent, will minimize the leaching of bioactive components during laundering while simultaneously imparting durable multifunctional properties to cotton fabrics. The synthesized dye was characterized by FTIR, ^1^H-NMR, ^13^C-NMR, and UV–Vis spectroscopy before being applied to cotton fabric through the exhaust dyeing process. The resulting textiles were evaluated for antibacterial, antifungal, antiviral, ultraviolet-protection, dye fixation, and laundering durability properties. The proposed one-step dyeing strategy integrates coloration and bioactivity into a single process and therefore has the potential to reduce processing steps, chemical consumption and wastewater generation compared with conventional sequential dyeing and antimicrobial finishing [13]. These potential environmental benefits are discussed qualitatively in this work; however, a comprehensive environmental life-cycle assessment was beyond the scope of the present study. Collectively, this work introduces a chloroxylenol-based vinyl sulfone reactive dye that combines durable coloration with antibacterial, antifungal, antiviral, and ultraviolet-protective functionalities through covalent fixation onto cotton fibers, thereby providing a promising strategy for the development of sustainable multifunctional bioactive textiles. As summarized in Table 1, conventional antibacterial dyes generally exhibit moderate fixation efficiency and limited durability, whereas the present chloroxylenol–VS system demonstrates enhanced dye fixation, prolonged antibacterial performance after repeated laundering, and additional multifunctional behavior including antifungal, antiviral, and UV-protective properties.

## 2. Experimental

### 2.1. Materials

Scoured and bleached cotton fabric (150 g m^−2^) was procured from a local textile mill. Para-ester (purity ≥ 98%) and sodium nitrite (NaNO_2_, purity ≥ 99%) were purchased from UNI-CHEM (Osaka, Japan), whereas chloroxylenol (purity ≥ 99%) was obtained from TCI Chemicals (Tokyo, Japan). Sodium carbonate (purity ≥ 99%), sodium hydroxide (purity ≥ 98%), hydrochloric acid (37%), sodium chloride (purity ≥ 99.5%), and all other chemicals and solvents of analytical grade were used without further purification unless otherwise stated. Deionized water was used throughout the synthesis and dyeing procedures. Gram-negative *Escherichia coli* (ATCC 25922) and Gram-positive *Staphylococcus aureus* (ATCC 6538) were obtained as KWIK-STIK™ Plus reference cultures from Microbiologics, Inc., St. Cloud, MN, USA, through MediMark^®^ Europe, Grenoble, France. *Aspergillus niger* cultures were kindly provided by the Atta-Ur-Rahman School of Applied Biosciences (ASAB), National University of Sciences and Technology (NUST), Islamabad, Pakistan, while Vero-E6 cells used for antiviral studies were obtained from the Cell Culture Bank, ASAB, NUST. A comprehensive experimental workflow summarizing the synthesis, dyeing, and characterization procedures is presented in Figure S1.

### 2.2. Synthesis of Functional Reactive Dye

#### 2.2.1. Diazotization of Para-Ester

**Figure 1:**
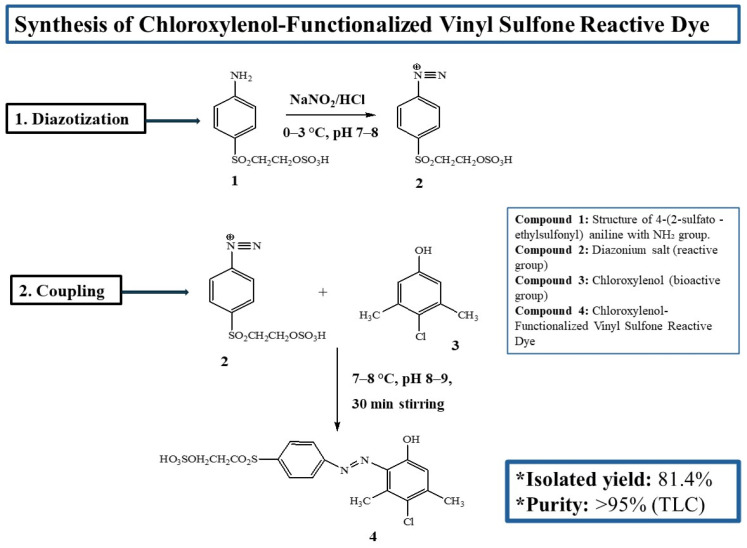
Reaction scheme for the synthesis of reactive dye from raw materials. * represent the synthesized reactive dye (4).

Para-ester (5.62 g, 0.02 mol; purity ≥ 99%, UNI-CHEM), as shown in Figure 1, was dissolved in 100 mL of distilled water at room temperature, and the pH was adjusted to 7–8 using 1 M NaOH under continuous stirring. The reaction mixture was cooled to 0 ± 1 °C using crushed ice (approximately 30 g), followed by the addition of 15 mL hydrochloric acid to generate the corresponding amine salt. A freshly prepared sodium nitrite solution (1.38 g, 0.02 mol in 20 mL distilled water) was then added dropwise while maintaining the temperature at 0 ± 1 °C under vigorous stirring. After completion of diazotization, excess nitrite was destroyed by adding sulphamic acid until starch–iodide paper showed no color change, confirming complete removal of residual nitrite and stabilization of the diazonium intermediate (mixture 2).

#### 2.2.2. Coupling of Chloroxylenol with Diazotized Para-Ester

Chloroxylenol (3) (3.1 g, 0.02 mol; purity ≥ 98%, TCI Chemicals, Japan) was dissolved in 20 mL of acetone under continuous stirring, and the pH of the solution was adjusted to 9–10 using 1 M NaOH. The reaction mixture was cooled to 0–3 °C using an ice bath. Subsequently, the diazotized para-ester (2) solution was added dropwise while maintaining the temperature at 7–8 °C and the pH between 8 and 9 through controlled addition of 1 M NaOH. After complete addition, the reaction mixture was stirred for 30 min, and the progress of the coupling reaction was monitored until a negative diazo/coupler ratio was obtained, indicating complete consumption of the diazonium salt.

The crude dye solution was then filtered, washed thoroughly with cold distilled water and acetone to remove unreacted starting materials and impurities, and dried under vacuum at 60 °C. The synthesized reactive dye (4) was obtained as a dark reddish-brown crystalline powder with an isolated yield of 81.4%. The reaction conditions were optimized through preliminary experiments by evaluating coupling completion and color development. The purity of the synthesized dye was confirmed by thin-layer chromatography (TLC) using silica gel 60 F254 plates with an ethyl acetate-based mobile phase, showing a single spot, indicating a purity of >95%. The overall synthesis pathway is shown in Figure 1.

**Figure 2:**
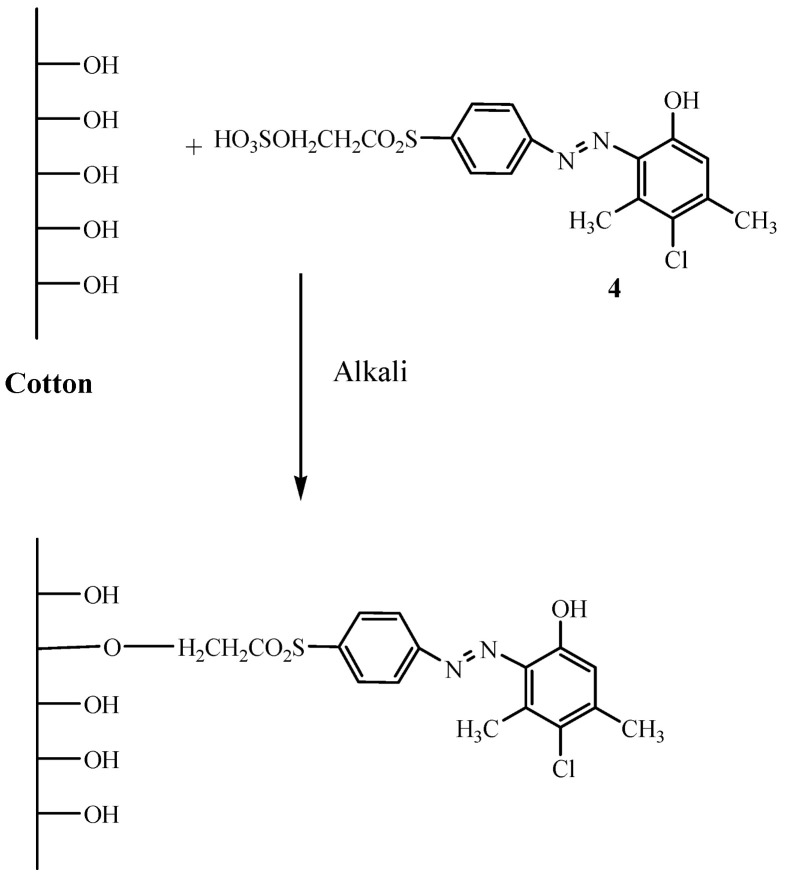
Covalent linkage formation between the hydroxyl group of cotton fabric and reactive dye.

It should be noted that the synthesized dye was isolated in its commercially stable β-sulfatoethylsulfone (SES) precursor form, as illustrated in Figure 1. During alkaline dyeing in the presence of sodium carbonate, the β-sulfatoethylsulfone group undergoes base-catalyzed elimination of sulfate to generate the corresponding vinyl sulfone reactive species in situ. It is this vinyl sulfone intermediate that subsequently reacts with the hydroxyl groups of cellulose to form the covalent dye–fiber bond. Therefore, Figure 1 and Figure 2 intentionally depict the stable precursor and the reactive form, respectively, which represent two successive stages of the reactive dyeing process.

## 3. Characterization of Synthesized Dye

The progress of the reaction and the purity of the synthesized dye were monitored by thin-layer chromatography (TLC) using aluminum-backed silica gel 60 F_254_ plates (Merck) with an ethyl acetate/methanol/water (70:25:5, *v*/*v*/*v*) developing solvent system. Upon completion of the coupling reaction, the crude dye was precipitated by the addition of a saturated sodium chloride solution and stirred for 30 min. The precipitated product was collected by vacuum filtration, washed repeatedly with cold distilled water until a neutral pH was attained, rinsed with a small amount of ethanol to remove residual organic impurities, and dried in a vacuum oven at 50–60 °C to a constant weight. The synthesized dye was obtained as a dark reddish-brown powder with an isolated yield of 81.4%. TLC analysis showed a single spot Rf = 0.71, indicating high purity (>95%).

The chemical structure of the dye was confirmed by ^1^H ^13^C nuclear magnetic resonance (NMR) spectroscopy (Bruker Avance, 600 MHz (Ettlingen, Germany)) using D_2_O as the solvent and sodium 3-(trimethylsilyl)propionate-d4 (TSP) as the internal reference standard. Fourier-transform infrared (FTIR) spectra were recorded on a PerkinElmer Spectrum, scan range 4000–500 cm^−1^, 32 scans, 4 cm^−1^ resolution. FTIR spectrometer to identify characteristic functional groups. UV–visible absorption spectra were obtained using a PerkinElmer Lambda 950 spectrophotometer to determine the maximum absorption wavelength (λ_max). The melting point was measured using a digital capillary melting point apparatus, while ultraviolet-protection factor (UPF) values were evaluated using a Transmittance Analyzer (UV-2000).

### 3.1. Dyeing Procedure, Color Strength, and Washing Fastness Measurement

#### 3.1.1. Dyeing Process

The HT-12E H-T TSUJII dyeing machine was used for the dyeing process. The exhaust process and synthetic reactive dye were used to dye the bleached and mercerized cotton cloth. The fabric was dyed with a 2% dye shade using a 1:50 liquor ratio.

Initially, the fabric was introduced into the dye bath at 25 °C and allowed to equilibrate for 10 min. Sodium chloride was subsequently added to promote dye exhaustion, and dyeing was continued for an additional 30 min. After a further 40 min, soda ash was introduced to facilitate fixation. The temperature was gradually increased from 40 to 80 °C and maintained for 45 min. Following dyeing, the fabric was rinsed thoroughly and soaped using 1 g L^−1^ standard detergent at 95 °C for 10 min to remove unfixed dye. The samples were then washed with distilled water and dried. The covalent linkage formed between cellulose hydroxyl groups and the reactive dye is illustrated in Figure 2.

#### 3.1.2. Dye Exhaustion

Dye exhaustion was determined spectrophotometrically by measuring the dye concentration in the dye bath before and after dyeing at λ_max = 535 nm using a UV–visible spectrophotometer. The percentage exhaustion (%E) was calculated according to Equation (1):

%E= [1 − *C*_2_/*C*_1_] × 100 (1)

where C1 and C2 represent dye concentrations before dyeing and after dyeing.

#### 3.1.3. Dye Fixation

A colored cotton sample was refluxed with 50% DMF for ten minutes to extract unfixed dye from the fabric’s surface in order to evaluate dye fixing. Next, a UV-Vis spectrophotometer was used to determine the dye concentration in the extract, while the following equation was used to estimate the percentage fixation (%F):

%F = [*C*_1_ − *C*_2_ − *C*_3_/*C*_1_ − *C*_2_] × 100 (2)

%T = (*%E* × *%F*) × 100 (3)

In this case, C3 represents the extracted dye concentration. Using percentage fixation (%F) and percentage exhaustion (%E), the total fixation of dye (%T) was computed using the following equation:

#### 3.1.4. Fastness Properties Evaluation

ISO standard methods were employed for the determination of fastness properties of dyed fabric: ISO 105-C06 for washing ((washing fastness test No. A2S or equivalent: 40 °C, 30 min, with standard detergent and 90g steel balls), ISO 105-X12 for rubbing (dry and wet, 10 cycles at 9 N force using a crockmeter with 16 mm finger), and ISO 105-B02 for light fastness (color fastness to artificial light using xenon arc lamp).

#### 3.1.5. Estimation of Leached Dye Concentration in Laundering Discharge

The amount of leached dye into water during the washing of fabric was estimated using the UV-Visible spectroscopic technique. Spectral analysis was conducted at (λ_max_) for a pure solution of the dye used for dyeing of cotton fabric. The fabric was washed for 10 washing cycles, and laundry discharge was collected. The washed fabric was subjected again to 10 more washing cycles, and the drain was collected. The UV-Visible absorbance spectra of the drain collected after 10 and 20 washing cycles were measured.

### 3.2. Functional Properties

#### 3.2.1. Minimum Inhibitory Concentration (MIC) Determination of Synthesized Dye

The minimum inhibitory concentration (MIC) of the synthesized dye was determined using a modified antimicrobial gradient method [15]. Dye concentrations of 5, 10, 20, 30, and 40 mg were individually dispersed in test tubes containing 9 mL of nutrient broth inoculated with *Staphylococcus aureus* (2 × 10^7^ CFU mL^−1^). An inoculated broth without dye served as the control. The suspensions were mixed using a vortex mixer for 5 min and incubated at 37 °C for 24 h. Following incubation, aliquots (45 μL) from each tube were spread onto nutrient agar plates and incubated again at 37 °C for 24 h. The MIC value was identified as the lowest dye concentration that completely inhibited bacterial growth. The same procedure was repeated for *Escherichia coli*.

#### 3.2.2. Qualitative and Quantitative Antibacterial Assessment

Both qualitative and quantitative assessments of dyed samples were carried out as described. The disk diffusion method, also known as the testing method AATCC-147, was used to conduct a qualitative evaluation of samples. Circular disks of fabric samples that had previously been autoclaved for 15 min at 121 °C were placed on a 25 mL agar plate that had been infected with a *S. aureus* bacterial culture. The zone of inhibition was then determined using the following relationship after sample-loaded agar plates were incubated at 37 °C for 24 h:

(4) $$Zone\;of\;inhibition\;\left(mm\right)=\frac{D2-D1}{2}$$

In this case, D2 is the diameter of the circular area where no bacterial colonial growth was seen, and D1 is the diameter of the sample disk.

The ISO 20743:2013 transfer method was used to perform the quantitative evaluation of antibacterial activity. For this, Gram-positive bacteria *S. aureus* and Gram-negative bacteria *E. coli* were employed. The relevant samples (control, dyed unwashed, and dyed washed cloth) were put on agar plates that had been injected with 1 milliliter of bacterial culture. For one minute, samples were compressed using a 200 g cylindrical weight. Samples were removed from the agar surface after one minute, placed upside down on an empty Petri dish, and incubated for 18 h at 37 °C. Following a 24 h incubation period, the fabric samples were collected in 20 milliliters of saline solution, vortexed for one minute, and serially diluted to 104. After preparing agar plates and spreading 60 µL of each dilution over the agar surface, all of the plates were set aside for incubation. At zero hour, the identical process was used. The incubation was not carried out once the bacteria had passed through contact with the fabric in order to conduct the experiment at 0 h. Standard deviations were computed after the testing was conducted twice. Using the following formulae, the log reduction (in terms of activity A) and percentage reduction were calculated by counting the number of bacterial colonies in logarithmic value of colony-forming unit per milliliter (CFU/mL) following inoculation at 0 and 18 h.

%Age Reduction = [(*log Ct* − *log Tt)*/*log Ct*] × 100 (5)

A = F − G (6)

where A is the antibacterial activity value, C0 and T0 are the bacterial counts of the control and treated fabric at 0 h, respectively, and Ct and Tt are the bacterial counts of the control and treated sample after 24 h, respectively. F = (log Ct − logC0) and G = (log Tt − log T0).

### 3.3. Antifungal Assessment

#### Quantitative Antifungal Assay

For quantitative antifungal evaluation, the AATCC 30 method was followed. The control and dyed samples were loaded on agar plates previously inoculated with fungal culture (1 × 10^5^) and the fabric was pressed down by applying a 200 g weight. Then, samples were removed from the agar surface, placed in empty Petri dishes and incubated at 25 °C for 72 h. The experiment did not use this incubation stage at 0 h. Following a 72 h incubation period, the samples were swirled in PBS and serially diluted up to 106. Each sample’s 50 µL dilution was streaked on agar plates, which were then incubated at 25 °C. Results have been reported on the third day after the identical process was carried out at 0 h. The following formula was used to determine the percentage reduction after counting the quantity of spores in the control and dyed washed fabrics.

(7) $$Percentage\;Reduction\;\left(\%\right)=\frac{A-B}{A}\times 100$$

where A and B are the number of spores on the control cotton fabric and dyed, washed fabric, respectively.

### 3.4. Antiviral Assessment

#### 3.4.1. Preparation of Infected Vero-E6 Cell Cultures

Dulbecco’s Modified Eagle Medium (DMEM) supplemented with 10% fetal bovine serum (FBS) and 1% penicillin–streptomycin–amphotericin B (PSA) was used to cultivate Vero-E6 host cells (obtained from the Cell Culture Bank). To generate viral stocks, confluent monolayers of Vero-E6 cells were inoculated with coronavirus (SARS-CoV-2 strain) at a dilution ratio of 1:4 in plastic culture flasks.

The development of viral cytopathic effects (CPEs) was monitored daily via light microscopy. Upon observation of 50–75% CPEs, the infected cultures were supplemented with 10% FBS and subjected to a single freeze–thaw cycle at −80 °C to lyse the host cells and release the viral particles. The cellular lysate was then subjected to low-speed centrifugation at 3500 rpm for 30 min at 4–8 °C to pellet cellular debris. The virus-containing supernatant was collected, aliquoted, and stored as the working viral stock for subsequent antiviral assays. To determine the viral titer, Vero-E6 cells were seeded in 96-well plates at a density of 2 × 10^4^ per cell and incubated at 37 °C under 5% CO_2_ for 24 h to achieve confluence. Ten-fold serial dilutions of the coronavirus stock (ranging from 10^−1^ to 10^−9^) were prepared. Confluent Vero-E6 monolayers were inoculated with each dilution and incubated for three days at 37 °C under 5% CO_2_. The 50% tissue culture infectious dose TCID50 or viral titer was calculated using the Behrens–Kärber method.

#### 3.4.2. Antiviral Activity Tests of Treated and Control Fabrics

Antiviral performance was evaluated using Vero-E6 cells seeded at a density of 2 × 10^4^ cells per well in 96-well plates. Fabric specimens (20 mm × 20 mm) were exposed to viral suspensions, and the filtrates obtained after contact with the textile samples were serially diluted from 10^−1^ to 10^−9^. The diluted suspensions were subsequently inoculated onto Vero-E6 cells and incubated at 37 °C under 5% CO_2_ for three days. Viral titers were calculated using the Behrens–Kärber method. Untreated cotton fabric was used as the control.

### 3.5. Cytotoxicity Evaluation

The cytocompatibility of the developed bioactive textile system was assessed using mouse fibroblast cells according to ISO 10993-5. Cell viability was determined using the Cell Counting Kit-8 (CCK-8) assay. Fibroblast cells were exposed to the extracts obtained from the dyed fabrics, and metabolic activity was measured spectrophotometrically. Cell viability values were calculated relative to untreated control cells, and the results were expressed as mean ± standard deviation.

### 3.6. Ultraviolet-Protection Factor (UPF) Measurement Evaluation

The ultraviolet-protection performance of the dyed cotton fabrics was evaluated according to the AATCC Test Method 183:2014 using a UV-2000F ultraviolet transmittance analyzer (Labsphere Inc., North Sutton, NH 03260, USA). Prior to measurement, fabric samples were conditioned under standard atmospheric conditions (21 ± 1 °C and 65 ± 2% relative humidity) for 24 h. Measurements were carried out over the wavelength range of 280–400 nm, covering both the UV-B (280–315 nm) and UV-A (315–400 nm) regions. Five different locations were selected randomly on each specimen to minimize the influence of fabric non-uniformity, and the corresponding UPF values were recorded. The reported UPF value for each sample represents the arithmetic mean of the five measurements. In addition to the UPF values, the percentages of UV-A and UV-B blocking were also calculated from the spectral transmittance data. Dyed unwashed fabrics and fabrics subjected to 20 industrial washing cycles were analyzed under identical conditions to evaluate the effect of laundering on ultraviolet-protection performance. All measurements were performed in triplicate and the results are presented as mean ± standard deviation.

## 4. Results and Discussion

### 4.1. FTIR

**Figure 3:**
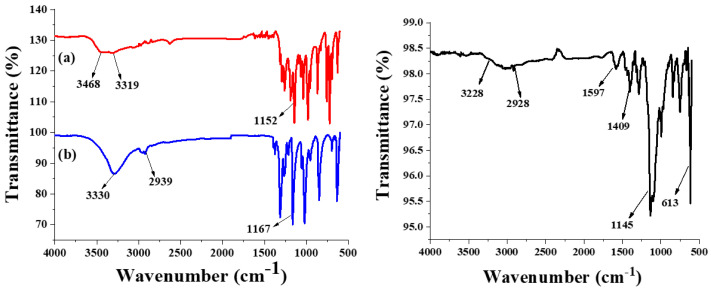
FTIR spectra of reactants (a) para-ester and (b) chloroxylenol (**left**) and synthesized reactive dye (**right**).

The FTIR spectra of para-ester (a), chloroxylenol (b), and the synthesized reactive dye (Figure 3 right) are presented in Figure 3. Comparison of the spectra reveals significant changes after diazotization and coupling, confirming the successful formation of the target azo reactive dye. The most noticeable spectral variations are observed in the regions of 3200–3500 cm^−1^ and 1400–1600 cm^−1^. In the spectrum of para-ester (Figure 3 left, two absorption bands located at 3468 and 3319 cm^−1^ are attributed to the asymmetric and symmetric stretching vibrations of the primary amino group (–NH_2_), respectively, which are characteristic features of aromatic amines [16]. A band observed at 1152 cm^−1^ corresponds to C–N stretching vibrations of the aromatic amine group. The spectrum of chloroxylenol exhibits a broad absorption band centered at 3330 cm^−1^, arising from O–H stretching vibrations of the phenolic hydroxyl group. The absorption at 2939 cm^−1^ is assigned to aromatic methyl (–CH_3_) C–H stretching vibrations, whereas the band at 1167 cm^−1^ is associated with C–O stretching of the phenolic moiety [17].

The FTIR spectrum of the synthesized dye (Figure 3 right) displays substantial differences from those of the starting materials, indicating successful conversion into the azo product. Notably, the disappearance of the two characteristic amino stretching bands at 3468 and 3319 cm^−1^ demonstrates the consumption of the primary amine group during diazotization and subsequent azo coupling. A broad absorption band appearing at 3228 cm^−1^ is assigned to hydrogen-bonded phenolic O–H stretching vibrations. The absorption peak at 1597 cm^−1^ is attributed to aromatic C=C skeletal vibrations, while the appearance of a new band at 1409 cm^−1^ is characteristic of the azo linkage (–N=N–) stretching vibration, providing direct evidence for the formation of the azo chromophore [18]. In addition, the band at 2928 cm^−1^ is assigned to aliphatic C–H stretching vibrations of methyl groups, whereas the strong absorption around 1145 cm^−1^ is associated with C–O stretching vibrations of the phenolic structure. The absorption band observed at 613 cm^−1^ corresponds to C–Cl stretching vibrations originating from the chlorinated aromatic ring of chloroxylenol [17]. These characteristic spectral changes collectively confirm the successful synthesis of the chloroxylenol-based vinyl sulfone reactive azo dye.

### 4.2. NMR Analysis

**Figure 4:**
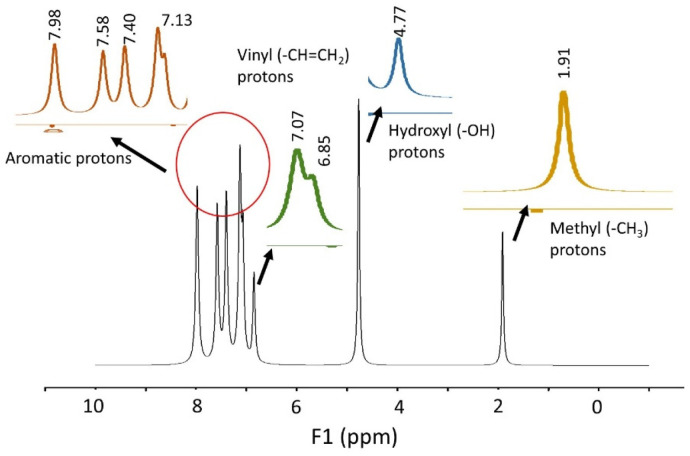
H^1^-NMR spectra of the synthesized bioactive reactive dye.

**Figure 5:**
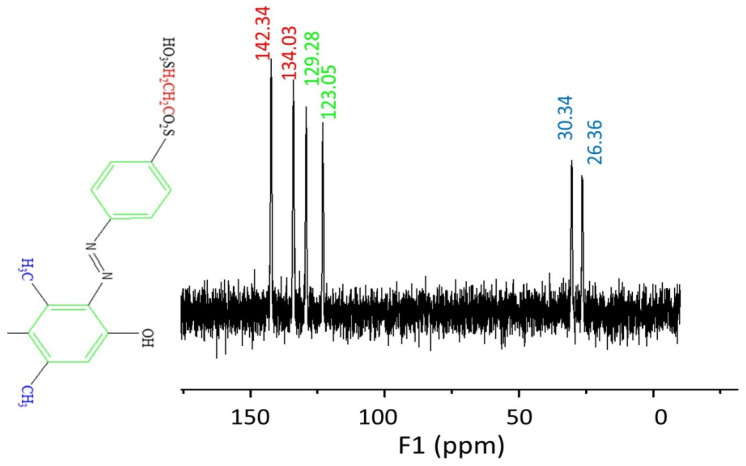
^13^C-NMR spectra of the synthesized bioactive reactive dye.

Both ^1^H and ^13^C NMR analyses were recorded using a Bruker Advanced 800 spectrophotometer (Bruker Optics, Ettlingen, Germany) running at 600 MHz. The isolated product was obtained in 81.4% yield and showed a purity of 95% (HPLC/elemental analysis), indicating successful synthesis and purification. The ^1^H NMR spectrum (600 MHz, D_2_O) exhibited characteristic resonances that are consistent with the proposed molecular structure (Figure 4). A singlet at δ 1.91 ppm integrating for six protons was assigned to the two equivalent methyl groups attached to the chloroxylenol aromatic ring. The chemical shift and integration pattern agree well with previously reported chloroxylenol-containing aromatic derivatives [19]. The vinyl sulfone moiety produced resonances in the δ 6.85–7.13 ppm region, corresponding to the vinylic protons (-CH=CH_2_). The appearance of these signals confirms the successful introduction of the reactive vinyl sulfone functionality, which is essential for covalent bond formation with textile substrates. Recent studies on vinyl sulfone reactive dyes similarly reported vinylic proton resonances in the aromatic/vinylic region between δ 6.5–7.5 ppm, supporting the present assignment [20,21]. A signal observed at δ 4.77 ppm was attributed to the phenolic hydroxyl proton associated with the chloroxylenol moiety. Furthermore, the multiple signals appearing within δ 7.13–7.98 ppm were assigned to aromatic protons belonging to the substituted benzene rings and azo-linked aromatic system [22]. Aromatic proton resonances in this region are characteristic of reactive azo dyes and have been extensively reported in NMR studies of sulfonated and bioactive azo dye structures [23]. The ^13^C NMR spectrum (600 MHz, CDCl_3_) provided additional confirmation of the molecular framework (Figure 5). The signals at δ 26.36 and 30.34 ppm were assigned to the methyl carbons attached to the chloroxylenol ring. Resonances at δ 123.05 and 129.28 ppm corresponded to aromatic sp^2^ carbons of the substituted phenyl rings [24,25]. The characteristic signals observed at δ 134.03 and 142.34 ppm were assigned to the two olefinic carbons of the vinyl sulfone group. The downfield position of these carbons is consistent with the electron-withdrawing effect of the sulfone functionality and agrees with reported NMR data for vinyl sulfone-containing reactive dyes [21]. The simultaneous observation of methyl, aromatic, hydroxyl, and vinyl sulfone resonances in both ^1^H and ^13^C NMR spectra provides strong evidence for the successful synthesis of the targeted bioactive reactive dye. In particular, the presence of the characteristic vinyl sulfone proton and carbon signals confirms preservation of the reactive functionality after synthesis. Such structural verification through detailed NMR peak assignment is considered essential for reactive dye characterization and reproducibility [26]. Complete ^1^H and ^13^C NMR peak assignments are provided in Table S1.

### 4.3. Concentration Assessment of Leached Dye in Laundry Discharge

**Figure 6:**
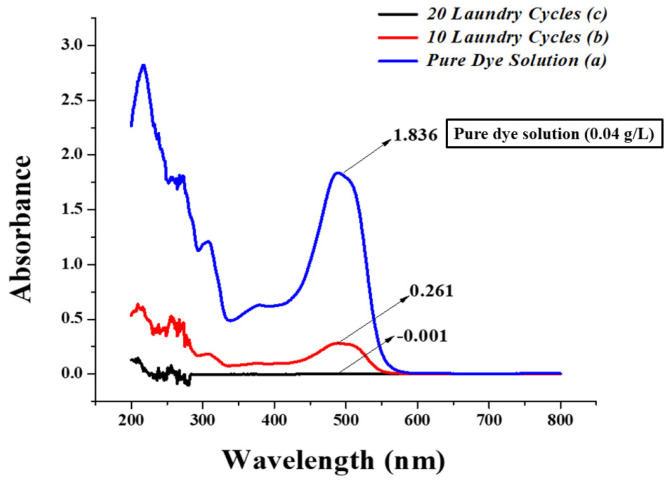
UV-Visible spectra of pure dye solution (a), laundry water after 10 (b) and 20 (c) washing cycles.

Concentration assessment of leached dye from fabric during wash was done using UV-Visible spectra for pure dye solution (a), laundry drain water collected after 10 launderings (b), and after 20 laundering cycles (c), as given in Figure 6. For the reactive dye solution, the maximum absorbance was observed at 505 nm. The leached dye concentration from the fabric surface during washing into laundering discharge was determined through a decrease in the peak intensity of spectra **b** and spectra **c** in comparison to the peak intensity of spectra **a**. The value of absorbance for laundry drain after 10 washing cycles and 20 wash cycles has been remarkably decreased from 1.836 (absorbance shown for pure solution of dye) to 0.261 and −0.001, which revealed that dye has not leached from the fabric surface while washing, confirming the durability of dye on fabric up to 20 industrial (equal to 100 home laundry) cycles. The absorbance value of spectra b (laundry drain after 10 wash cycles) was slightly higher than that of spectra c (laundry drain after 20 wash cycles), which could be due to unfixed dye removal present on the fabric surface during the early phase of washing. Since the chloroxylenol (bioactive agent) is covalently linked to the dye moiety, as confirmed by ^1^H-NMR and ^13^C-NMR characterization, the dye non-leaching behavior corroborates that chloroxylenol, being an integral constituent of the dye structure, has also not leached out into the discharge during laundry. The environmental advantage of the developed system originates primarily from the covalent immobilization of chloroxylenol within the reactive dye structure. Conventional antimicrobial finishes often release active substances during laundering, contributing to the discharge of bioactive compounds into wastewater streams. In contrast, the negligible dye release observed after repeated washing suggests enhanced retention of the active component on the textile substrate. Therefore, rather than claiming complete environmental safety, the present results indicate a reduced potential for chemical release during use, which may contribute to lowering the environmental burden associated with antimicrobial textile finishing processes [27].

### 4.4. Dyeing Performance and Fixation Behavior

**Figure 7:**
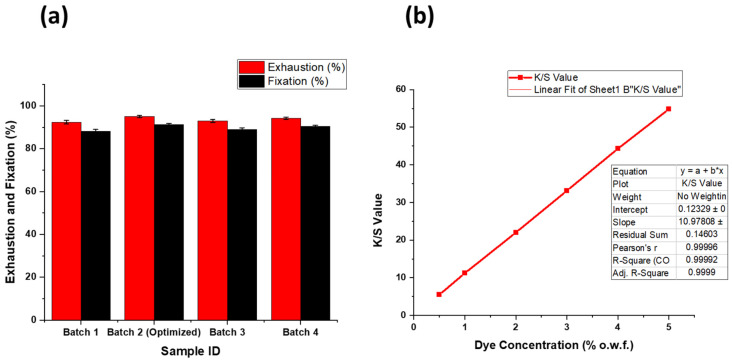
Assessment of the dyeing behavior and colorimetric properties of the prepared reactive dye on cotton fabric: (**a**) dye exhaustion and fixation efficiencies at four independent experimental batches (error bars are used to indicate standard deviation, *n* = 3); (**b**) color strength (K/S) development with respect to dye concentration (0.5 to 5.0 percent o.w.f.), with a linear regression.

Figure 7a,b systematically studied the dyeing properties and fixation behavior of the produced reactive dye to investigate its practical applications and chemical efficiency. According to Figure 7a, the exhaustion and fixation efficiencies have been estimated with four separate experimental batches. The dye had high substantivity to cellulosic fibers, and exhaustion values exceeded 92%, while the highest exhaustion value reached about 95.1% for Batch 2. In alkaline dyeing, the structural removal of the precursor produces the reactive vinyl sulfone group and attaches the covalent bond to the hydroxyl groups of the fiber, thus producing the upper fixation efficiency up to 91.2%. The difference between exhaustion and fixation values indicates that the amount of dye hydrolysis is minimal, thus reducing the amount of unreacted dye and increasing the environmental efficiency of the process. Additionally, the low standard deviation of the results of all batches indicates the high reproducibility of the dyeing process. Figure 7b depicts the effect of dye concentration (0.5–5.0% o.w.f.) on the color strength (K/S). It can be seen that there is a pronounced linear increase in K/S values when the dye concentration increases up to 54.8 for 5.0% o.w.f. The good linearity proves that there is a direct proportionality of the dye concentration to the color strength, as indicated by a coefficient of determination (R^2^ = 0.99992). This indicates that the number of active sites in the fiber was not saturated; therefore, efficient dye adsorption occurred even at such a high dye concentration. Consequently, deeper colors can be obtained without any loss of dye adsorption efficiency. Additionally, high color strength, excellent exhaustion, and fixation efficiencies demonstrate that the proposed dyeing system is highly suitable for high-performance textile applications.

### 4.5. Antibacterial Activity and Optical Density (OD) Analysis

**Figure 8:**
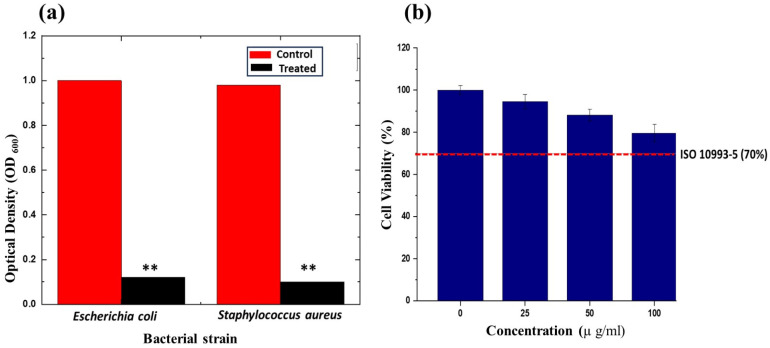
(**a**) Comparison of optical density (OD_600_) of *E. coli* and *S. aureus* following exposure to control and dyed fabric extracts. The degree of turbidity in the treated groups decreased significantly (*p* < 0.01, denoted by **), which is equivalent to about 88–92% inhibition of bacterial growth. (**b**) Evaluation of the bioactive textiles in terms of cytotoxicity.

The quantitative assessments of the antibacterial activity of the chloroxylenol-functionalized fabric were done in liquid culture by measuring optical density (OD_600_) as indicated in Figure 8a. Strong bacterial growth in the control groups of *Escherichia coli* and *Staphylococcus aureus* was observed, with an OD_600_ value of 1.00 and 0.98, respectively. Conversely, the functionalized fabric extracts treatment led to a substantial decrease in bacterial growth (*p* < 0.01). OD_600_ values fell to 0.12 in the case of *E. coli* and 0.10 in the case of *S. aureus*, which represents about 88–92 percent growth. These findings are in line with the agar diffusion assays and they validate the high antibacterial activity of the treated fabric. The large decrease in bacterial biomass of both Gram-negative and Gram-positive bacteria indicates the wide-spectrum efficacy and consistent antimicrobial activity of the chloroxylenol-functionalized textile. The significant reduction in the OD values indicates the high broad-spectrum antibacterial activity of the covalently immobilized chloroxylenol bioactive agent on both bacterial strains used.

### 4.6. Cytotoxicity Assessment

L929 mouse fibroblast cells (ATCC^®^ CCL-1™) were obtained from the American Type Culture Collection (ATCC), Manassas, VA, USA. The L929 cell line is a continuous murine fibroblast cell line recommended by ISO 10993-5 for the in vitro evaluation of cytotoxicity of biomaterials. The MTT assay with L929 fibroblasts was used as a method of assessing the biocompatibility of the bioactive textile system, and the results are shown in Figure 8b. The viability of the cells was high in all the concentrations tested, which confirmed the high safety profile of the immobilized antimicrobial agent. Cell viability of 25 and 50 µg/mL was 94.7% and 88.2%, respectively, with no significant effect on cellular metabolic activity. Viability was approximately 80 at the highest concentration of 100 µg/mL, exceeding the 70% threshold of ISO 10993-5 of non-cytotoxic material. This outstanding cytocompatibility has been largely attributed to the fact that chloroxylenol is immobilized effectively in the dye structure. Unlike traditional antimicrobial finish systems, which rely on a leaching action and can lead to localized toxicity, the release of free active agent is limited in this system. Consequently, antimicrobial activity is maintained on the fabric surface, and the negative impacts on human cells are also reduced. Altogether, the produced bioactive textile has a good balance between antimicrobial functionality and biological safety, which allows its use in long-term contact with the skin. The results are reported in terms of the mean of triplicate experiments. The MTT assay was used to determine the cell viability of L929 fibroblasts after 24 h incubation with various concentrations of fabric extract (0–100 µg/mL). The red dashed line represents the 70% viability level required by ISO 10993-5; values exceeding this level are non-cytotoxic. The data are presented as mean ± standard deviation (*n* = 3).

### 4.7. Minimum Inhibitory Concentration (MIC) Determination of Synthesized Dye

**Figure 9:**
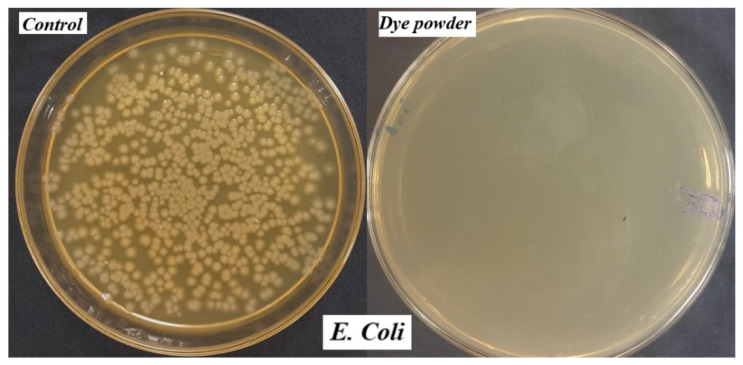
Antibacterial activity (MIC) of synthesized dye powder against *E. coli*.

**Figure 10:**
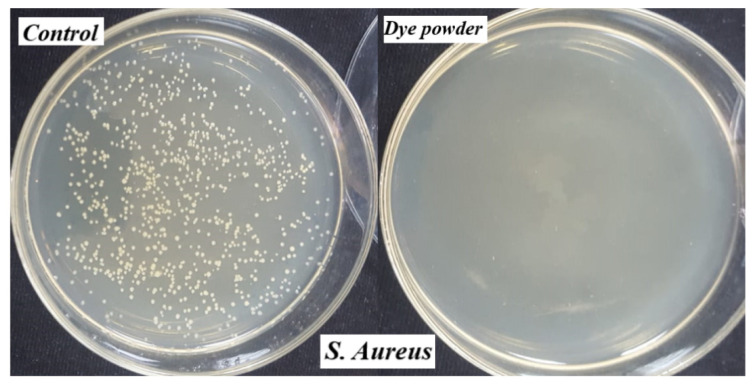
Antibacterial activity (MIC) of synthesized dye powder against *S. aureus*.

Minimum inhibitory concentration of dye against both *S. aureus* and *E. coli* was evaluated. The obtained results have been summarized in Table 2. It was observed from the values that the MIC of the as-synthesized dye is 10 and 20 mg for *S. aureus* (Gram-positive) and *E. coli* (Gram-negative) bacterial strains, respectively. The dye powder exhibited 99.99% bactericidal activity at these concentrations for both bacterial strains (*E. coli* and *S. aureus*), as shown in Figure 9 and Figure 10. No significant reduction in bacterial colony growth was observed at these concentrations. It was observed that the dye concentration required for 99.99% reduction in *E. coli* was higher than that of *S. aureus*. The higher dye concentration required to achieve complete inhibition of *E. coli* compared with *S. aureus* can be attributed to differences in bacterial envelope architecture rather than cell-wall thickness alone. Gram-positive bacteria such as *S. aureus* possess a thick peptidoglycan layer but lack an outer membrane, allowing antimicrobial agents to interact more readily with the cytoplasmic membrane. In contrast, Gram-negative bacteria such as *E. coli* contain an additional outer membrane composed of lipopolysaccharides that acts as an effective permeability barrier and restricts the diffusion of many antimicrobial compounds. Consequently, Gram-negative bacteria generally exhibit greater intrinsic resistance to antimicrobial agents than Gram-positive bacteria, requiring higher concentrations to achieve equivalent bactericidal effects [28].

### 4.8. Qualitative Antibacterial Assessment

**Figure 11:**
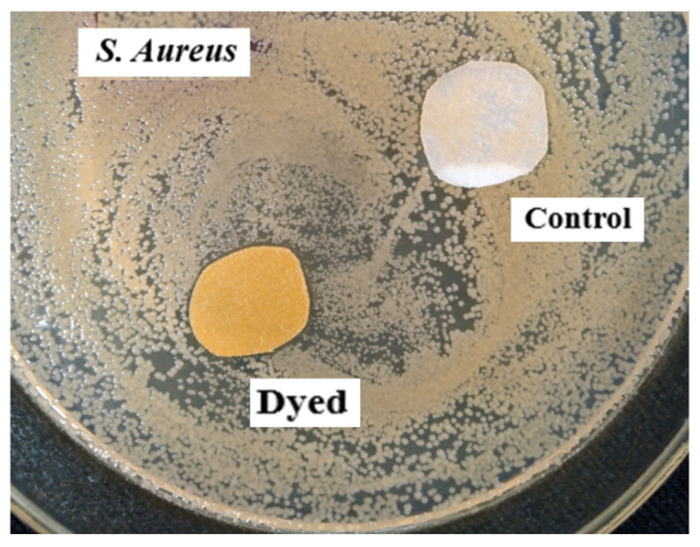
Qualitative antibacterial activity of the dyed fabric and undyed cotton fabric (control), indicating non-leaching behavior of bioactive agent (chloroxylenol).

To check the antibacterial potency of dyed fabric, the samples were tested against the bacterial strain (*S. aureus*) according to the standard disk diffusion method (AATCC 147), and the obtained results are given in Figure 11. This figure shows the antibacterial activity of the dyed cotton fabric after 20 washing cycles and the raw cotton (control) fabric. It can be seen clearly from the picture that no significant zone was present around the dyed fabric, but no significant bacterial colony growth was observed below and above the fabric surface. There is also no bacterial growth around the dyed fabric in its vicinity, as can be seen from the thin lining around the fabric, where no bacterial colonies are present. The absence of ZOI and no bacterial growth above, below and around the dyed fabric confirmed that the bioactive agent applied on the fabric is non-leaching. The non-leaching bioactive agents act through a contact mechanism, i.e., they do not peel off from the fabric surface into the agar medium to show their activity, but they do kill the microbes when they come in contact with the fabric (treated with bon-leaching bioactive agents) surface. On the contrary, enormous colonies of bacteria were observed below and above the control fabric surface, which confirmed that the antibacterial activity shown by the dyed fabric is due to the applied bioactive dye. The dyed fabric showed activity even after going through 20 industrial wash cycles, which established that our developed dyed bioactive textiles are sustainable as these textiles are durable, reusable and do not leach the applied antibacterial agent into the environment through laundry drain during washing.

### 4.9. Quantitative Antibacterial Analysis

The ISO-20743 transfer method was followed to carry out quantitative antibacterial analysis of dyed cotton fabric (both washed and unwashed). The antibacterial potential of the tested samples was evaluated and expressed in terms of log reduction and percentage reduction using the formula given in the methodology section, and the obtained results have been summarized in Table 3. The dyed fabric has shown excellent antibacterial activity against *S. aureus* and *E. coli*, killing 99.99% and 94.66% of inoculated bacterial colonies, respectively. The antibacterial activity of dyed washed fabric has also revealed outstanding antibacterial activity, i.e., exhibited 96.36% and 91.36% bactericidal action against *S. aureus* and *E. coli* bacteria. The potential antibacterial action exhibited by dyed, washed fabric confirmed excellent durability of dyed bioactive cotton fabric, which means activity is sustained even after excessive laundering cycles, establishing the non-leaching effect of chloroxylenol. The excellent durability to washing has also confirmed that the antibacterial agent chloroxylenol has successfully integrated chemically into the dye structure and is not leached from the fabric at the laundry stage, which was the major objective of our research work. The outstanding durability of dyed cotton fabric is due to the fact that chloroxylenol is covalently immobilized within the dye; the antimicrobial action is expected to occur predominantly through contact-mediated interactions rather than through release of active molecules into the surrounding medium. Previous studies suggest that phenolic compounds may disrupt membrane integrity, alter membrane permeability and inhibit enzyme activity, ultimately causing microbial inactivation. Nevertheless, the exact mechanism associated with immobilized chloroxylenol species was not directly investigated in the present study and therefore requires further experimental verification [29]. The high fixation rates (>90%) of vinyl-sulfone-based reactive dyes are due to the formation of a strong covalent linkage between the vinyl group (-CH=CH_2_) of the reactive dye and hydroxyl (-OH) groups of cotton fabric.

The excellent antibacterial action of dyed bioactive fabric could be ascribed to the chloroxylenol integrated into the structure of the dye. The previous literature has reported strong antimicrobial efficacy of chloroxylenol against several broad-spectrum Gram-negative and Gram-positive bacteria [30]. The possible mechanism of antibacterial activity of chloroxylenol involves its attachment to the surface of bacteria, followed by penetration into the bacterial cell wall, and disruption of essential cellular components required for bacterial survival, ultimately resulting in cell death.

Some other reported reasons for its antibacterial action involve the deactivation of enzymes, immobilization of essential proteins and modification in cell-membrane permeability, inhibiting the coupling between phosphorylation reactions and electron transport, which eventually inhibits ATP synthesis [31].

**Figure 12:**
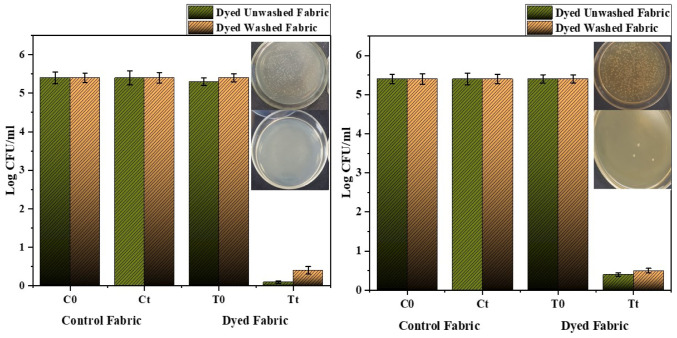
Antibacterial activity (log CFU/mL) of dyed unwashed and dyed washed fabric against *S. aureus* (**left**) and *E.coli* (**right**).

The antibacterial potential for washed and unwashed dyed fabric samples against bacterial strains *E. coli* and *S. aureus*, as log of CFU/mL, is shown in Figure 12. Where C_0_ and C_t_ represent the control log (CFU/mL) and T_0_ and T_t_ correspond to the log (CFU/mL) for unwashed fabric and washed fabric (at 0 h and 24 h), respectively. This figure shows the growth of bacterial colonies and the number of surviving colonies after 24 h, expressed as log counts, for all tested samples (control, dyed washed, and dyed unwashed). A remarkable decrease in log values was observed for unwashed dyed fabric (5.42 to 0) and washed dyed fabric (5.42 to 0.30) against *S. aureus* after 24 h, while in the case of control fabric, the log value was found to be increased (5.42 to 5.45) after 24 h, suggesting no antibacterial activity in control fabric. Likewise, the log value also reduced significantly for the unwashed dyed sample (5.44 to 0.30) and the washed dyed sample (5.44 to 0.47) against *E. coli* after 24 h. The results indicated that the antibacterial action of dyed cotton fabric was higher (0, 99.99%) for *S. aureus* as compared to *E. coli* (0.30, 96%). The slightly higher antibacterial activity observed against *S. aureus* than against *E. coli* is consistent with the structural differences between Gram-positive and Gram-negative bacteria. Although Gram-positive bacteria possess a thicker peptidoglycan layer, they lack the outer membrane characteristic of Gram-negative bacteria. The outer membrane of *E. coli* contains lipopolysaccharides and functions as an additional permeability barrier, limiting the penetration of antimicrobial molecules into the bacterial cell. Therefore, Gram-negative bacteria generally demonstrate greater resistance to antimicrobial agents, which may explain the slightly lower bacterial reduction observed against *E. coli* in the present study [28].

### 4.10. Quantitative Antifungal Analysis

**Figure 13:**
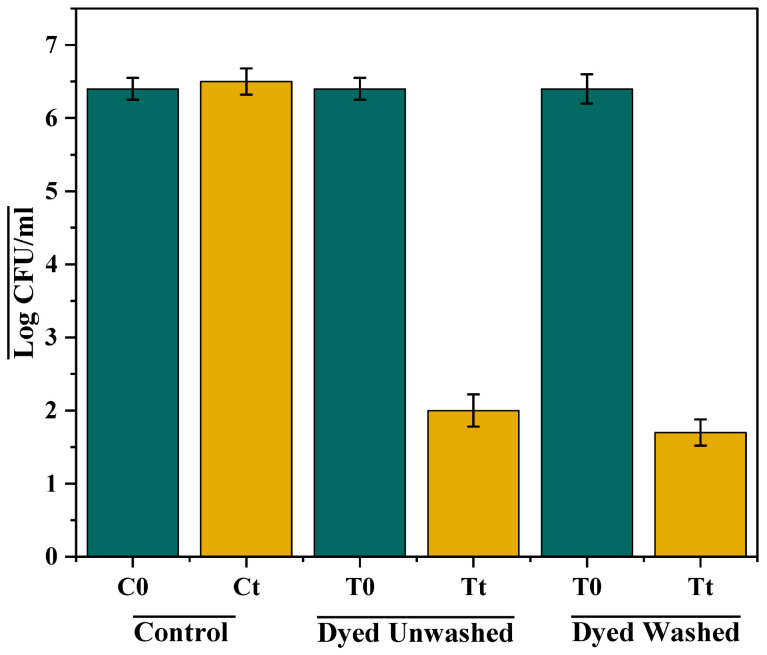
Antifungal activity (log SFU/mL) for dyed unwashed and washed fabric.

To conduct the quantitative evaluation of antifungal activity, the AATCC 100-2004 standard quantitative method was followed, and *Aspergillus niger* was used for this purpose. The control cotton fabric (C), dyed unwashed fabric, and dyed fabric after 20 washing cycles were tested. All samples were incubated for 0 h and 72 h. The number of spores was counted to calculate the percentage reduction in the tested samples. The dyed unwashed fabric washed fabric has revealed 86% (1.96 log10) antifungal activity against *A. niger*, while the washed fabric showed 82% (1.68 log 10) activity, as can be seen from Figure 13. The antifungal activity of the dyed cotton fabric was retained even after 20 industrial laundering cycles, indicating strong covalent fixation of the reactive dye onto the cellulose matrix. Such durable bonding minimizes leaching of the chloroxylenol-containing dye during washing and contributes to long-lasting bioactivity. The proposed mechanism of chloroxylenol for its antifungal property could be related to its molecular structure. The non-polar (benzene ring) part of chloroxylenol penetrates the membrane, causing delocalization of electrons, resulting in membrane destabilization [32]. The antifungal mechanism of the synthesized dye is most likely associated with the surface-exposed chloroxylenol moiety. Because the dye is chemically anchored to cotton, the bioactive phenolic group remains available at the fiber–fungus interface, where it can interact directly with fungal cells [33]. Chloroxylenol is known to disrupt membrane integrity by its hydrophobic aromatic structure, which can penetrate the lipid-rich fungal envelope and destabilize the plasma membrane. In addition, the phenolic hydroxyl group may interfere with membrane-bound proteins and enzyme systems, leading to leakage of cellular contents, impaired metabolism, and inhibition of spore germination [34]. The persistence of antifungal activity after 20 industrial laundering cycles suggests that the observed bioactivity originates from the chemically immobilized chloroxylenol moiety rather than from the release of antimicrobial species into the surrounding medium. Owing to the covalent fixation of the reactive dye onto cellulose, chloroxylenol functional groups remain exposed at the fiber surface, allowing direct interaction with fungal spores upon contact. The hydrophobic aromatic ring of chloroxylenol is believed to partition into lipid-rich membrane regions, resulting in increased membrane permeability and loss of membrane integrity. Simultaneously, the phenolic hydroxyl group can interfere with membrane-associated proteins and enzyme systems, causing leakage of intracellular constituents and inhibition of metabolic activity. Membrane disruption has been recognized as one of the most effective antifungal mechanisms because fungal membranes are essential for maintaining cellular homeostasis and hyphal growth [35]. Furthermore, damage to the plasma membrane may impair ergosterol-dependent functions and inhibit spore germination, thereby suppressing the proliferation of *Aspergillus niger*. Therefore, the retained antifungal activity after repeated washing supports a durable contact-active mechanism arising from the surface-accessible chloroxylenol groups anchored within the reactive dye structure.

### 4.11. Antiviral Analysis

**Figure 14:**
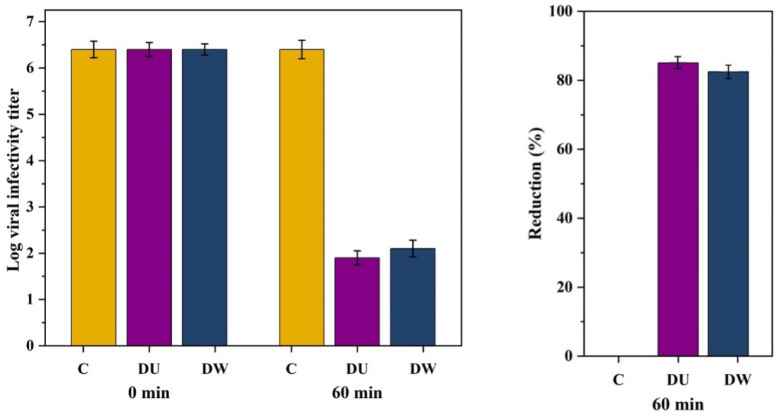
Log viral infectivity titer and percentage reduction in control (C), dyed unwashed (DU), and dyed washed (DW) fabric.

The antiviral performance of the chloroxylenol-functionalized cotton fabrics was evaluated against SARS-CoV-2 by monitoring changes in viral infectivity after 60 min of contact (Figure 14). Viral infectivity titers were determined using the Behrens–Kärber method. As shown in Figure 14, the untreated cotton fabric exhibited no measurable antiviral activity under the experimental conditions. In contrast, the dyed unwashed and dyed washed fabrics achieved reductions in viral infectivity of 87% and 83%, respectively, after 60 min of contact. Although these reductions correspond to less than a 1-log reduction in viral titer, they demonstrate measurable antiviral activity that was largely retained following 20 industrial laundering cycles. The relatively small decrease in antiviral performance after repeated washing suggests good durability of the functionalized textile under the investigated laundering conditions. The precise antiviral mechanism of the chloroxylenol-functionalized reactive dye was not directly investigated in the present study. Based on the previous literature, phenolic compounds may reduce the infectivity of enveloped viruses through interactions with the viral lipid envelope and surface glycoproteins, thereby impairing viral attachment, membrane fusion, or other early stages of viral infection [36]. Chloroxylenol (4-chloro-3,5-dimethylphenol) has previously been reported to exhibit antiviral activity against enveloped viruses, and its phenolic structure is capable of interacting with biological membranes and viral proteins [37]. In the present work, the retention of antiviral activity after repeated laundering is consistent with the durable performance of the reactive dye–cotton system under the investigated conditions. However, the contribution of chloroxylenol immobilization to the observed antiviral activity was not directly examined, and neither quantitative chloroxylenol release nor detailed antiviral mechanistic studies were performed. Therefore, the proposed mechanism should be regarded as a plausible interpretation based on previous literature rather than direct experimental evidence from this study. Previous studies have suggested that phenolic compounds can alter the structural integrity of viral envelopes by disrupting hydrogen-bonding interactions within viral proteins, leading to conformational changes that reduce viral infectivity [38]. Nevertheless, the molecular interactions responsible for the antiviral activity of the present chloroxylenol-functionalized reactive dye remain to be elucidated through dedicated mechanistic investigations. Future studies employing direct analyses of antiviral pathways together with quantitative leaching assessments would provide a more comprehensive understanding of the relationship between dye immobilization, durability, and antiviral performance.

### 4.12. Ultraviolet-Protection Factor (UPF) Measurement

**Figure 15:**
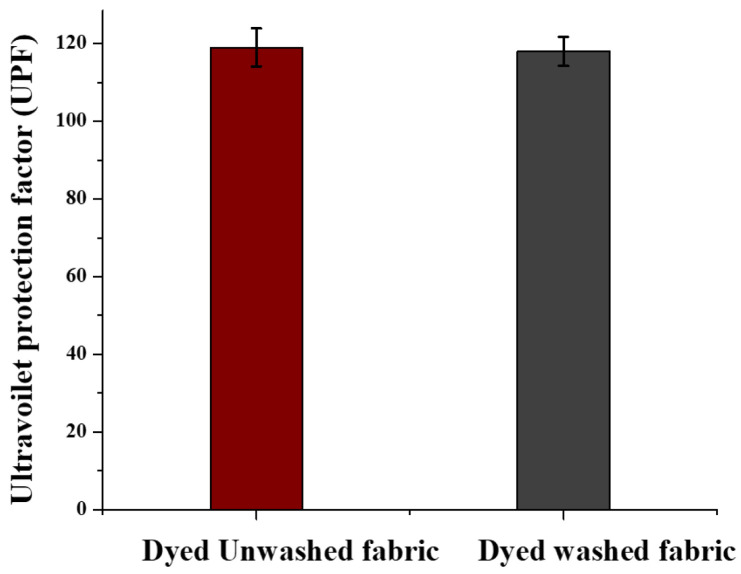
Ultraviolet-protection factor (UPF) mean values of dyed unwashed and dyed washed fabric: error bars are the standard error of the mean.

The ultraviolet-protection performance of the fabrics was determined according to AATCC 183:2014. Measurements were conducted over the wavelength range of 280–400 nm at five randomly selected locations on each specimen, and the reported values represent the mean ± standard deviation of triplicate measurements. The scanning was performed over the wavelength range of 280 nm to 400 nm; however, the average across all measured UPF values was used as the fabric’s UPF rate. Figure 15 shows the measured UPF values (mean) and their standard deviations for both washed and unwashed dyed samples. The figure clarifies the UPF values for both samples to be greater than 100, which means excellent UV protection [39]. The dyed unwashed fabric shows the highest value for UPF, about 119, and a slight decline is observed in the UPF value after 20 wash cycles to 118, which could be related to the removal of dye that remained unfixed on the fabric surface at the dyeing stage of cotton and thus removed during early cycles of fabric washing.

**Figure 16:**
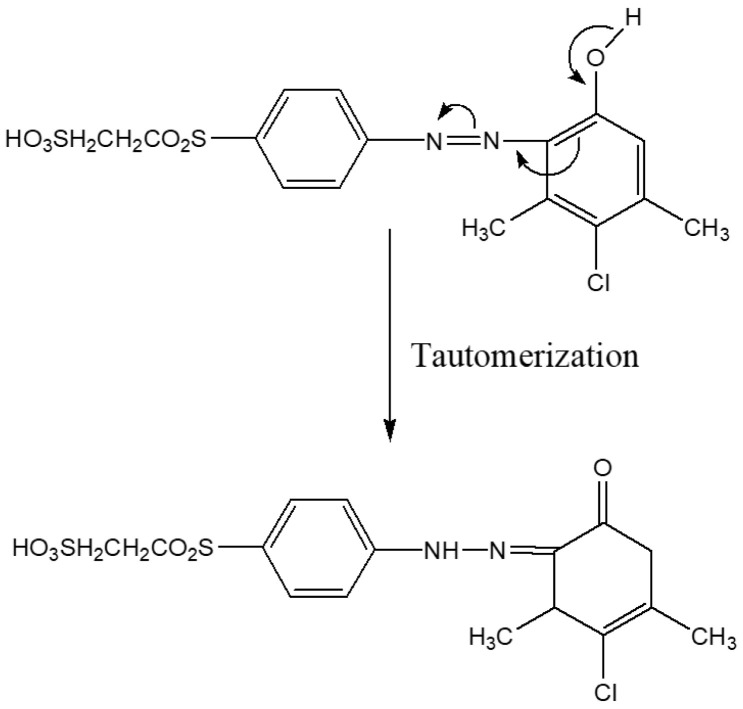
Tautomerization phenomenon occurring in synthesized bioactive reactive dye.

The excellent UPF value could be related to the presence of auxochromic groups (-OH, -NHR) present inside the dye moiety. These groups are responsible for the strongest UV absorption (harmful radiation) and for releasing thermal energy into the ambient environment or converting it into long-wavelength radiation. In addition, the azo group present in the synthesized dye shows conjugation with the hydroxyl moiety and undergoes the phenomenon of tautomerism (Figure 16), responsible for the strongest UV absorption. The high energy associated with these rays is then utilized in the tautomer formation, whereby excessive energy is released in the form of heat [40].

**Figure 17:**
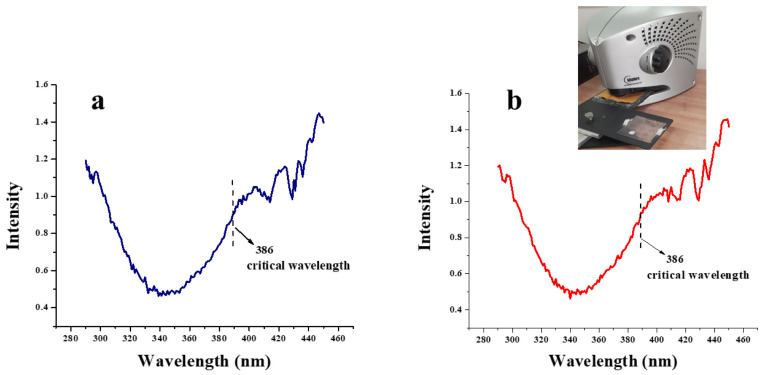
UV-transmittance spectra in terms of UPF values of dyed fabric unwashed and (**a**) after 20 washing cycles (**b**).

UV absorption is shown in Figure 17a,b for both dyed, unwashed, and washed samples. Both fabric types show the strongest absorption in the UVA and UVB regions. Both samples show a critical wavelength of about 386 nm (ideal wavelength for fabric having good UPF, which infiltrates only 10% of ultraviolet rays) and, thus, are considered the best samples having excellent UV protection. In addition, with an increase in critical wavelength value from 370 nm, it indicates that UV protection functions in a broader spectrum.

The UPF values for five different locations are summarized in Table 4, which also shows the percentage blocking of samples against UV rays in both regions (UVA and UVB). From the table, it can be seen that unwashed dyed samples blocked 99.35% of UVA and UVB rays, respectively. Similarly, the percentage blockage shown by washed dyed samples were 98.98% and 98.97% for both UVA and UVB rays after 20 wash cycles. However, the mean UPF value measured for washed and unwashed dyed fabric is 119 and 118, respectively. It can be deduced from the given results that dyed washed fabric exhibits excellent protection properties against UV rays, which is sustained even after 20 industrial washes.

### 4.13. Wash Durability and Stability of Biological Activity

**Figure 18:**
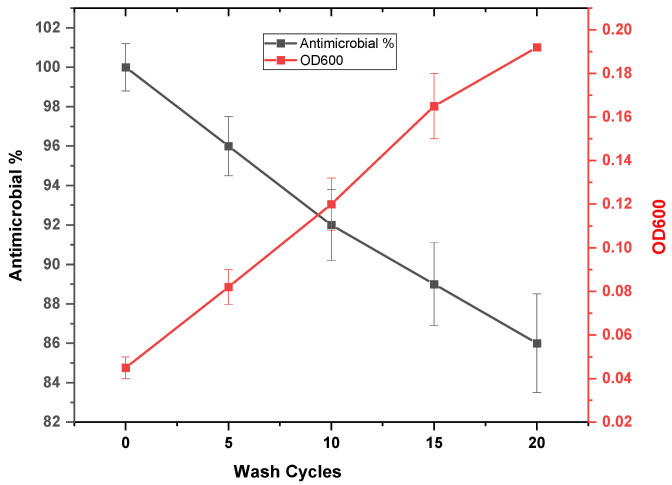
Durability and biological stability of functionalized bioactive textiles after 20 wash cycles. The antimicrobial activity (black, primary Y-axis) is above 86% and bacterial growth (red, secondary Y-axis; OD_600_) is below 0.20 for the repeated laundering, confirming a high stability of the functional finish. Covalent immobilization using vinyl sulfone chemistry leads to improved durability. Values are expressed as mean ± SD (*n* = 3).

To assess the mechanical and chemical stability of the used antimicrobial finish, the laundered functionalized textile underwent 20 accelerated wash cycles (Figure 18). The findings show that bioactivity is well preserved, and the antimicrobial activity (black profile) does not drop below 86% even with the highest number of laundering cycles. Bacterial growth kinetics support this high stability pattern, with the optical density (OD_600_) values (red profile) gradually increasing with the entire range of washing, 0.04–0.19. Notably, the (OD_600_) values are lower than the critical microbial growth level of 0.20, ensuring that the antimicrobial-treated textile has the necessary antimicrobial activity to prevent the growth of microorganisms during repeated laundering. The mechanistic basis of this sustained performance is the reactive vinyl sulfone-based chemistry, which facilitates the formation of stable covalent ether bonds between the dye-bioactive complex and the hydroxyl groups of the cellulosic substrate. Compared to traditional physically adsorbed or leaching-type finishes, in which active agents are quickly depleted during washing, the covalent immobilization approach guarantees permanent immobilization of the functional constituents. This results in the system having an impressive balance between laundering durability and long-lasting antimicrobial activity, suggesting it could be used in long-term biomedical and protective textile applications.

### 4.14. Fastness Properties

For examination of fastness properties like washing, rubbing and lightfastness, ISO standard methods (ISO 105-C06, ISO 105-X12, and ISO 105-B02) were used for cotton fabric, with results being given in Table 5. Excellent rubbing and washing fastness (4–5) were obtained for dyed fabric, whereas the light fastness obtained varies from moderate to good on grayscale (3–4). Results showed that fabric dyed with a synthesized novel reactive dye exhibited excellent fastness properties, and significantly high exhaustion (>90%) and fixation (>90%) rates were observed. These high fixation and exhaustion rates, along with fastness properties, could be due to the presence of the vinyl (-CH=CH_2_) reactive group in the dye structure, which forms a strong covalent bond with the cotton fabric hydroxyl groups.

### 4.15. Sustainability Considerations

The developed simultaneous dyeing and functional finishing approach presents significant environmental advantages over conventional two-step dyeing and finishing processes. The higher rates of dye fixation (91%) and exhaustion (95%) result in a significant reduction in the amounts of unfixed dye in the textile effluent compared to conventional post-finishing approaches, which often revealed lower fixation rates (60–80%). Moreover, UV-Vis analysis of laundry discharge further confirmed the negligible dye release after 20 industrial laundry cycles, which indicated a significant reduction in chemical discharge into textile wastewater. Also, the integrated approach eliminates separate post-finishing steps, thereby reducing water, energy, and chemical consumption. The literature on similar reactive dyeing strategies documented water efficiency of up to 50% and reduced CO_2_ emissions when high-fixation dyes were employed. Future work will include a complete cradle-to-gate LCA to quantify these benefits more comprehensively.

## 5. Conclusions

This study successfully developed a novel chloroxylenol-functionalized vinyl sulfone reactive dye capable of simultaneously imparting coloration and durable bioactivity to cotton fabric through a single-step dyeing process. Structural characterization by FTIR, UV–Vis, ^1^H-NMR, and ^13^C-NMR confirmed the successful synthesis of the target dye molecule. The reactive dye exhibited high dye exhaustion and fixation, together with excellent antibacterial, antiviral, antifungal, ultraviolet-protective, and colorfastness properties. The retention of these functional properties after repeated laundering demonstrates the durability of the developed textile system under the conditions investigated. Overall, the results indicate that the synthesized reactive dye is a promising candidate for the development of multifunctional cotton textiles requiring durable bioactive performance.

### 5.1. Key Findings

- Dye exhaustion: 95%.
- Dye fixation: 91%.
- MIC against *S. aureus*: 10 mg/9 mL.
- MIC against *E. coli*: 20 mg/9 m.
- Antibacterial activity before washing:
  ▪ A total of 99.99% against *S. aureus.*
  ▪ A total of 94% against *E. coli.*
- Antibacterial activity after 20 laundering cycles:
  ▪ A total of 96% against *S. aureus.*
  ▪ A total of 91% against *E. coli.*
- Antifungal activity:
  ▪ A total of 86% before washing.
  ▪ A total of 82% after 20 laundering cycles.
- Antiviral activity:
  ▪ A total of 87% before washing.
  ▪ A total of 83% after 20 laundering cycles.
- Ultraviolet-Protection Factor (UPF):
  ▪ A total of 119 before washing.
  ▪ A total of 118 after washing.
- UV blocking efficiency:
  ▪ A total of 99.35% UVA blockage.
  ▪ A total of 98.98% UVB blockage.
- Cell viability (MTT assay):
  ▪ Greater than 80% at 100 μg mL^−1^.
- Wash durability:
  ▪ Functional bioactivity is retained after 20 industrial laundering cycles.
- Fastness properties:
  ▪ Wash fastness: 4–5.
  ▪ Rubbing fastness: 4–5.
  ▪ Light fastness: 3–4.

### 5.2. Significance of the Study

The results demonstrate that incorporating chloroxylenol into a reactive dye structure provides an effective strategy for producing multifunctional cotton textiles with durable antibacterial, antiviral, antifungal, and ultraviolet-protective properties. Unlike conventional antimicrobial finishing systems that rely on surface deposition of active agents, the present approach integrates coloration and functionalization within a single dye molecule through covalent attachment to the textile substrate. The high dye fixation and retention of biological activity after 20 laundering cycles support the durability of the developed system under the experimental conditions evaluated in this study. These findings suggest that the proposed approach has potential for applications requiring durable multifunctional textile performance. However, comprehensive assessment of long-term environmental behavior, active-agent release under diverse use conditions, and biomedical performance requires further investigation.

### 5.3. Future Perspectives

The present study demonstrates the successful development of a chloroxylenol-functionalized vinyl sulfone reactive dye capable of imparting durable antibacterial, antifungal, antiviral, and ultraviolet-protective properties to cotton fabrics. Although the developed system exhibited excellent performance and wash durability, several aspects warrant further investigation. Future studies should focus on elucidating the molecular mechanisms responsible for microbial inactivation by the immobilized chloroxylenol moieties using techniques such as reactive oxygen species (ROS) analysis, membrane permeability assays, electron microscopy, and molecular docking simulations. These investigations would provide a more comprehensive understanding of the antimicrobial mechanism. In addition, surface analytical techniques including scanning electron microscopy (SEM), X-ray photoelectron spectroscopy (XPS), atomic force microscopy (AFM), and energy-dispersive spectroscopy (EDS) could be employed to investigate the morphology, elemental distribution, and chemical stability of the dye–fiber interface following repeated laundering and prolonged use. From an application perspective, future work should evaluate the performance of the developed dye on additional textile substrates, including polyester, viscose, bamboo, and blended fabrics, as well as its compatibility with industrial dyeing processes. Further studies should also investigate long-term biocompatibility, skin compatibility, and safety through appropriate in vitro and in vivo assessments before biomedical or prolonged wearable applications are considered. In addition, quantitative environmental assessments, including life-cycle analysis and studies of potential active-agent release under realistic service conditions, are needed to determine the overall sustainability of the proposed approach relative to conventional dyeing and finishing processes. Overall, the present work provides a foundation for the development of multifunctional reactive dyes with durable bioactive properties and highlights promising directions for future research aimed at advancing sustainable functional textile technologies.

## Figures and Tables

**Table 1:** Comparison of antibacterial reactive dye systems based on reactive chemistry, fixation efficiency, antimicrobial performance, durability, and multifunctional properties.

Dye System	Reactive Group	Fixation (%)	Antibacterial Reduction (%)	Durability (Washes)	Multifunctionality	Reference
**Sulfonamide-based**	Triazine/VS	70–85	90–95 (bacteria)	10–20	Antibacterial only	[13]
**Chloroxylenol–triazine**	Triazine	80–88	>95 (bacteria)	15–20	Antibacterial, UV	[14]
**Present work (Chloroxylenol-VS)**	Vinyl sulfone	91–95	99.99/94 (unwashed), 96/91 (after 20 laundry cycles)	20	Antibacterial, Antifungal, Antiviral, UV	Current Research

**Table 2:** MIC values of synthesized dye against and *E. coli* and *S. aureus*.

Sr #	Dye Concentration (mg/9 mL)	Percentage Reduction (%)	
		*S. aureus*	*E. coli*
1	5	25	13
2	10	99.99	31
3	20	99.99	99.99
4	30	99.99	99.99
5	40	99.99	99.99

**Table 3:** Log and percentage reduction values of samples against *S. aureus* and *E. coli*.

Sample	MIC (mg/9 mL)		Log Reduction (18 h)		Percentage Reduction (%)	
	*S. aureus*	*E. coli*	*S. aureus*	*E. coli*	*S. aureus*	*E. coli*
**Control (undyed)**	-	-	0.03	0.03	0	0
**Dyed Unwashed**	10	20	2.19	2.17	99.99	94
**Dyed Washed (20 cycles)**	10	20	2.18	2.19	96	91

Note: The values listed in above table indicate mean of replicates. ISO 20743 standard protocol is followed for the calculation of percentage (%) reduction. Higher antibacterial activity towards *S. aureus* (Gram-positive bacteria) is consistent with outer membrane differences in Gram-negative bacteria (*E. coli*).

**Table 4:** Ultraviolet-protection (UPF) measurement data of dyed fabric (unwashed and washed).

Sample	No. of Scans	UPF Value	Mean UPF Value	UV-A Blockage (%)	UV-B Blocking (%)
**Dyed fabric (unwashed)**	Scan 1	116.77	119	99.35	98.98
	Scan 2	128.07			
	Scan 3	117.90			
	Scan 4	116.87			
	Scan 5	117.12			
**Dyedfabric (washed)**	Scan 1	119.20	118	99.35	98.97
	Scan 2	119.01			
	Scan 3	112.24			
	Scan 4	122.47			
	Scan 5	118.71			

**Note:** The values listed in Table 3 are measured according to AATCC 183:2014 standard protocol 2014 (UV-2000F analyzer, 280–400 nm, 5 random locations, mean ± SD of triplicates). UPF > 80 suggests exceptional UV protection.

**Table 5:** Exhaustion, fixation, and fastness (rubbing, light, and washing) result from the dye.

Reactive Dye	Exhaustion %	Fixation %	Rubbing Fastness	Light Fastness	Wash Fastness
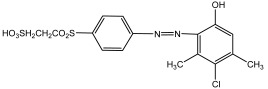	95	91	4–5	3–4	4–5

## Data Availability

The original contributions presented in this study are included in the article/Supplementary Material. Further inquiries can be directed to the corresponding author.

## Supplementary Materials

The following supporting information can be downloaded at: https://www.mdpi.com/article/10.3390/biomimetics11070477/s1, Figure S1: A comprehensive experimental flowchart summarizing the one-step synthesis, dyeing, and characterization workflow. Table S1: Complete ^1^H and ^13^C NMR peak assignments.
