# Supplementary material for: Embedding the Bioactive Agent in Dye Structure for Development of Environmentally Sustainable Bioactive Textiles
Source: Biomimetics (Basel). 2026 Jul 8;11(7):477. doi: 10.3390/biomimetics11070477 (PMC13406689; doi:10.3390/biomimetics11070477)
Supplement: Supplementary file 1 [file biomimetics-11-00477-s001.zip › biomimetics-4324686-supplementary.pdf]

List of Supplementary Figures

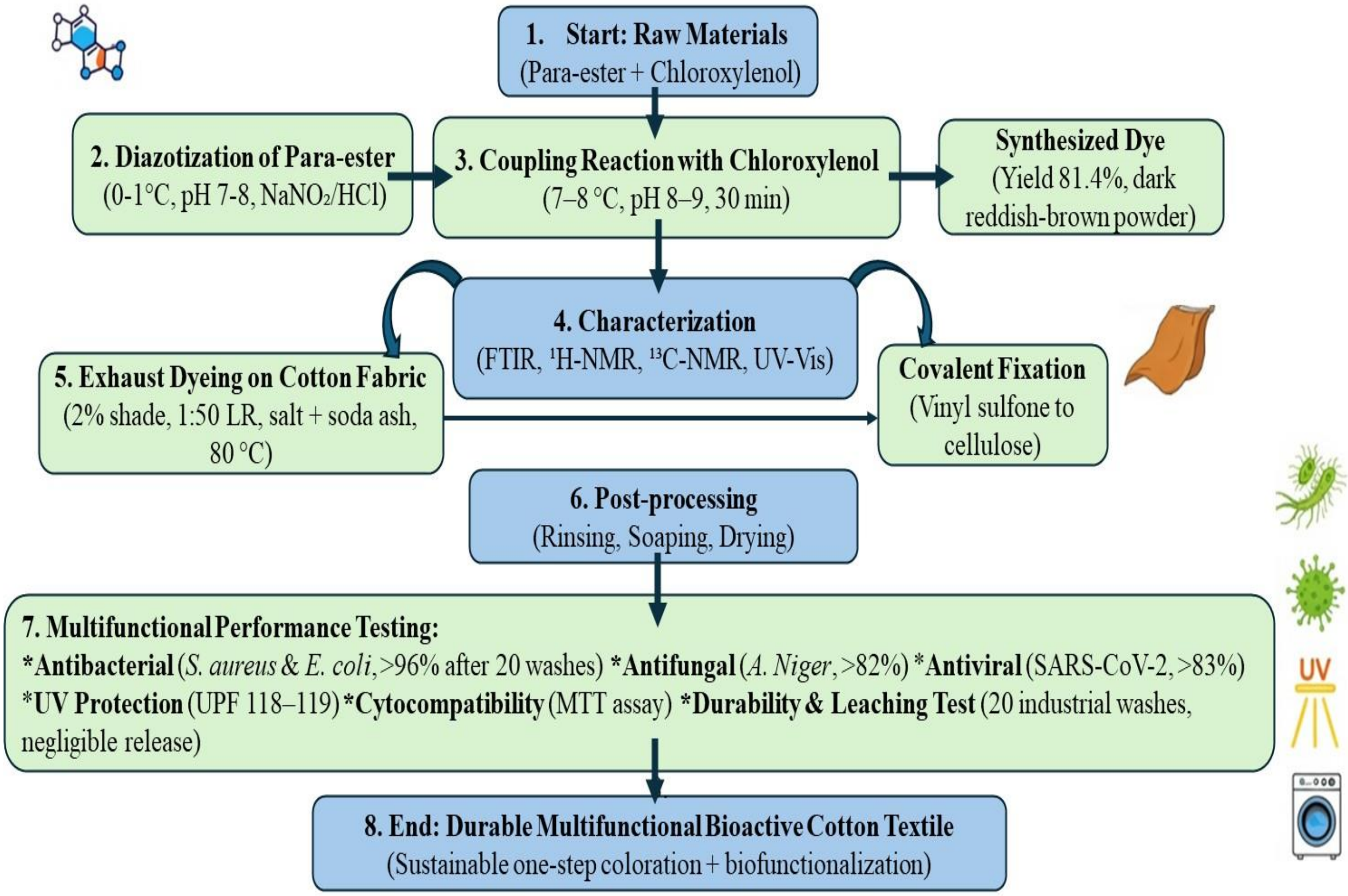

Figure S1: A comprehensive experimental flowchart summarizing the one-step synthesis, dyeing, and characterization workflow.

List of Supplementary Tables

Table S1 : Complete ^{1}H and ^{13}C NMR peak assignments

Nucleus	δ (ppm)	Assignment	Structural Fragment
1H	1.91	6H, s	2 × CH_3 attached to chloroxylenol ring
1H	4.77	1H, s	Phenolic OH
1H	6.85–7.13	3H	Vinyl sulfone (-CH=CH_2) protons
1H	7.13–7.98	m	Aromatic protons
13C	26.36	CH_3 carbon	Chloroxylenol methyl carbon
13C	30.34	CH_3 carbon	Chloroxylenol methyl carbon
13C	123.05	Aromatic carbon	Phenyl ring carbon
13C	129.28	Aromatic carbon	Phenyl ring carbon
13C	134.03	Vinyl carbon	-CH=CH_2
13C	142.34	Vinyl carbon adjacent to SO_2	-CH=CH_2
